# Toward a 3D physical model of diffusive polymer chains

**DOI:** 10.3389/fphy.2023.1142004

**Published:** 2023-06-29

**Authors:** Andras Karsai, Grace J. Cassidy, Aradhya P. Rajanala, Lixinhao Yang, Deniz Kerimoglu, James C. Gumbart, Harold D. Kim, Daniel I. Goldman

**Affiliations:** 1 School of Physics, Georgia Institute of Technology, Atlanta, GA, United States,; 2 School of Chemical and Biomolecular Engineering, Georgia Institute of Technology, Atlanta, GA, United States,; 3 School of Chemistry and Biochemistry, Georgia Institute of Technology, Atlanta, GA, United States

**Keywords:** granular media, polymer physics, experimental methods, fluidized beds, 3D printing, discrete element methods

## Abstract

Recent studies in polymer physics have created macro-scale analogs to solute microscopic polymer chains like DNA by inducing diffusive motion on a chain of beads. These bead chains have persistence lengths of O(10) links and undergo diffusive motion under random fluctuations like vibration. We present a bead chain model within a new stochastic forcing system: an air fluidizing bed of granular media. A chain of spherical 6 mm resin beads crimped onto silk thread are buffeted randomly by the multiphase flow of grains and low density rising air “bubbles”. We “thermalize” bead chains of various lengths at different fluidizing airflow rates, while X-ray imaging captures a projection of the chains’ dynamics within the media. With modern 3D printing techniques, we can better represent complex polymers by geometrically varying bead connections and their relative strength, e.g., mimicking the variable stiffness between adjacent nucleotide pairs of DNA. We also develop Discrete Element Method (DEM) simulations to study the 3D motion of the bead chain, where the bead chain is represented by simulated spherical particles connected by linear and angular spring-like bonds. In experiment, we find that the velocity distributions of the beads follow exponential distributions rather than the Gaussian distributions expected from polymers in solution. Through use of the DEM simulation, we find that this difference can likely be attributed to the distributions of the forces imparted onto the chain from the fluidized bed environment. We anticipate expanding this study in the future to explore a wide range of chain composition and confinement geometry, which will provide insights into the physics of large biopolymers.

## 1. Introduction

Recent macro-scale physical models of polymer chains and molecules in 2D use a variety of techniques to mimic the thermodynamic behavior of microscopic molecules on a macroscopic scale. A common theoretical model used to describe the dynamics of polymer is a “beads on a chain” model, in which point masses are linked together with a flexible element [1]. By analyzing the effects of uncorrelated noise on these model systems theoretically, conclusions can be made about the dynamics of polymer chains [2]. However, simplifications within theoretical models can hide complexities present in a physical system [3]. Experimental analogs prove a useful tool to investigate phenomena with complex environmental interactions. For instance, Safford et al. showed experimentally that the collective dynamics of externally vibrated granular chains are similar to Rouse models of polymers, where long chains are well modeled with a self avoiding random walk coupled with center of mass diffusion [4]. Reches et al. reproduced short chain RNA hairpins using electrostatically charged bead chains on a similar vibrating plate. Teflon and Nylon beads were given opposite charges and separated with smaller spacer beads to allow for the mobility required to form a hairpin [3]. Prentis and Sisan used a chain of ping pong balls to simulate a polymer; the chain was placed on a flat surface along with many self-propelled, motorized spheres to simulate solvent interactions [5]. These macroscopic models allow for insights into the dynamics of polymers that would be difficult to probe through purely theoretical or computational approaches.

One challenge in macro-scale studies is replicating the random Brownian motion found in polymer and solvent molecules. On a macroscopic scale, the system will reach static equilibrium unless a driving force is provided. Such driving force, however, may lead to a dynamic equilibrium that is fundamentally different from a thermodynamic equilibrium attained by microscopic systems. It is important to ensure that particles in these macro-scale models move with analogous velocity distributions to those found on a molecular level. Tricard et al. tracked the motion of a three-unit chain on a shaken plate with the addition of small spheres serving as gas molecule analogs. They found that the chain typically adopted one of three distinct conformations, occuring with probabilities following a Boltzmann distribution dependent on agitation frequency [6]. However, little work has been done to extend these 3D techniques for physical modeling of polymers to three dimensions.

To simulate the environment of a polymer, it is necessary to create randomly distributed forces in three dimensions. Fluidized beds supply such an environment, allowing for granular motion in three dimensions. When the drag force of a fluid moving upward through a bed of solid particles (generated by resistance due to flow through the porous medium) reaches a critical value relative to the downward force of gravity, the weight of the bed becomes equal to the pressure drop as a result of the fluid flow, transitioning the particles from a solid-like state to a fluid-like state as their averaged bulk movement is suspended in the fluid flow. Increasing the fluid flow further decreases the solids’ bulk density and increases their mean velocities [7].

Advances in 3D printing technology allow for the precise and rapid manufacture of finely detailed custom plastic parts at low cost. Such parts can be used for a wide variety of applications, such as biomedical devices, electronics, robotics, and jewelry [8, 9]. Liquid-Crystal Display (LCD) printing with a photo-curable resin as a low cost technology allowing for high resolution printing [8]. Commercial LCD technology can create parts on the scale of 250μm [9]. Since it is crucial to be able to control the density of the beads such that the chain is neutrally bouyant in the fluidized bed, the density of 3D printed parts can be modulated by setting the print’s fill density or by including cavity space in the printed model. In this paper, we seek to create the first 3D macroscale analog that describes polymer chain dynamics according to the “beads on a chain” model discussed above, through the combination of the two aforementioned technologies: fluidized beds and liquid-crystal display (LCD) resin printing.

## 2. Materials and equipment

### 2.1. Bead chain

**FIGURE 1:**
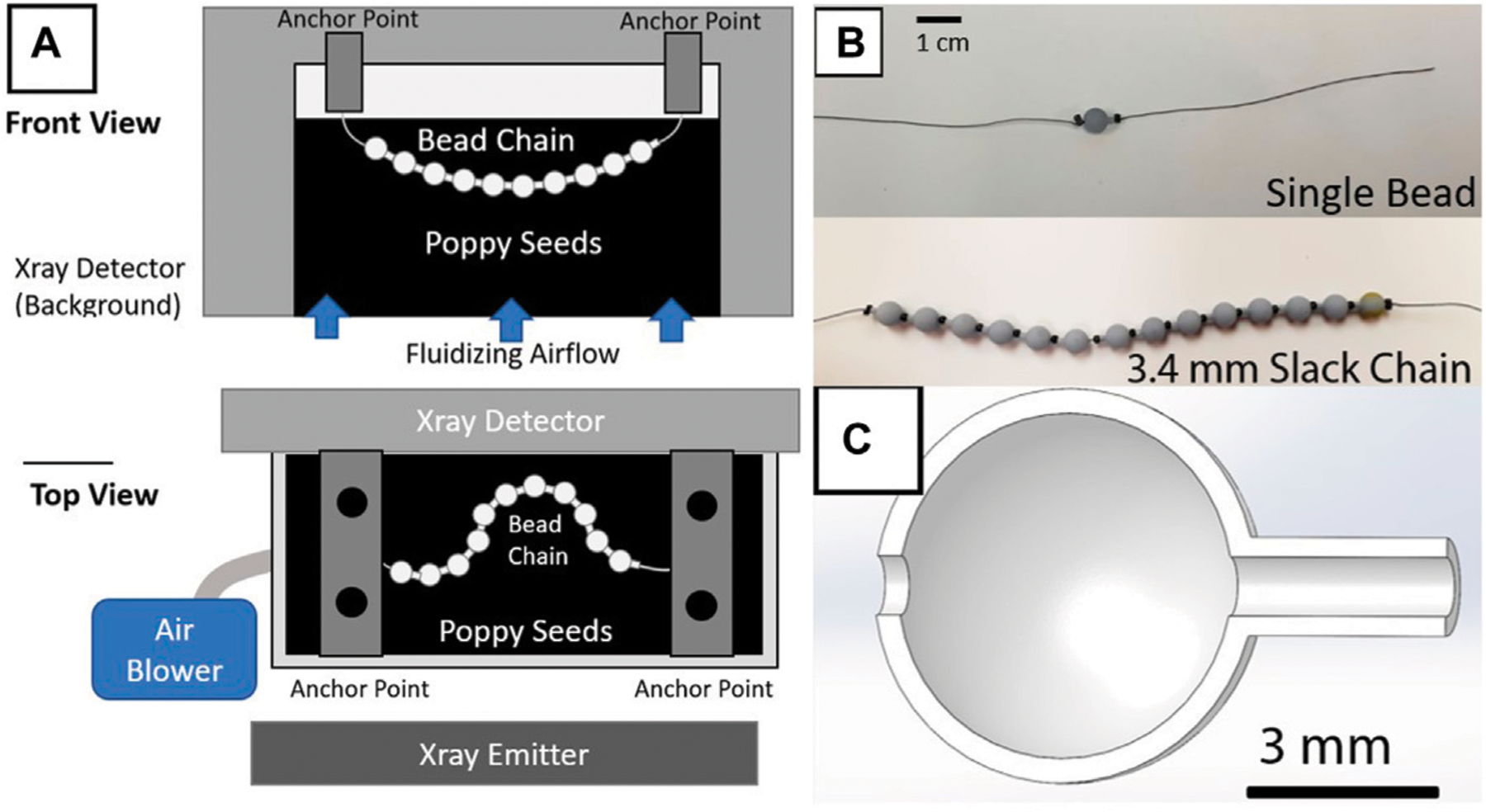
Experimental setup diagram and bead chain samples (**A**) Front view diagram of the experimental setup. A small acrylic bed filled with poppy seeds is placed between an X-ray emitter and a corresponding detector (Orthoscan DI Mini C-arm), with an air blower generating fluidization. The tested bead chain is then suspended from two metal bars spanning the bed to constrain its kinematics within the X-ray field of view. (**B**) Photos of the single bead and full chain configurations. (**C**) Cutaway of the 3D model for a single bead. The thread is guided through the center, with crimp beads trapping the resin bead in place along the string. Varying the infill of the beads’ cavities can modulate the chain’s effective density.

The bead chains in this study consist of 3D printed resin beads shaped into hollow spheres (diameter 6 mm) with a cylindrical protrusion (length 1.5 mm, diameter 1.5 mm) (Figures 1B, C). The thickness of the sphere’s walls is 0.4 mm. The sphere and protrusion are designed to mimic a basic ball-and-stick model of a polymer chain [1], with most of the mass of the bead concentrated in the spherical portion. Each bead includes a 1 mm diameter hole running along its length as seen in (Figure 1C). The beads are printed from Anycubic UV-activated resin in an LCD Photon Mono X resin printer. Cylindrical metal crimp beads (commonly used in jewelry making) measuring 2 mm in diameter and 1.4 mm in length are strung onto silk suture thread alternating with the resin bead units. The crimp beads on the ends of the chain are crimped with pliers to prevent beads from sliding off the ends of the string. The crimp beads in the middle of the chain are left uncrimped to provide additional rotational freedom and flexibility between resin bead units. The metal beads are radio-opaque, unlike the resin beads, allowing them to be imaged by X-rays while immersed in a optically opaque bed of particles.

Three bead-chains were used for the study, designated as a single resin unit chain, a “slack” chain, and a non-slack chain. To control the amount of slack introduced into the chain, the chain was held upright when the final crimp bead was locked in place, allowing the beads to compress vertically under gravity. The slack length is the length of bare string between the top of the highest resin bead and the bottom of the crimp bead. The non-slack chain had no slack such that adjacent crimps and beads were always in contact, and the slack chain had 3.4 mm of slack. Both the slack chain and the non-slack chain contain 14 resin beads alternating with 15 crimp beads. This resulted in a total length of approximately 12.5 cm for each chain, along with additional string on each end for attachment. The single-bead chain consisted of one central resin bead flanked by two crimp beads with no slack.

### 2.2. Fluidized bed

Poppy seeds [10] were agitated in an air-fluidized bed of size 22 cm by 10.8 cm by 11 cm. Poppy seeds are well suited to this application because they are relatively uniform in size and, in our tests, we observed they are more transparent to X-rays than other granular substances such as glass particles. The poppy seeds are overall monodisperse soft grains with a mean diameter of 1 mm and a bulk density of ϕ⋅ρ≈ 570 kg/m^3^, where ϕ=0.58 is the loosely packed volume fraction and ρ is the material density of poppy seeds. Air was forced upward through the bed using a Toro Ultra leaf blower (12 Amps/340 CFM maximum) connected to a plexiglass distribution chamber of about 5 cm in height. A Variac was used to manually vary the voltage to the blower thus controlling the air flow rate to achieve fluidizing conditions. Calculation of the minimum fluidization velocity $U_{f}$ with the Ergun equation [11] for our setup predicts $U_{f}\approx 0.32\;\text{m}/\text{s}$. An Omega hot-wire anemometer placed approximately 5 cm above the center of the bed measured air velocity and ensured consistent conditions across trials. Each trial used an airspeed velocity of U=0.55m/s to achieve fluidization, which is greater than $U_{f}$. An Orthoscan DI Mini C-arm X-ray captured video of the bead chain while it is immersed in the fluidized bed. A schematic of the bed is shown in Figure 1.

Two vertical bolts were secured 10 cm apart overhanging the bed roughly 1 cm above the surface of the bed to serve as attachment points for the chain. The chain was secured at each end in order to prevent it from leaving the field of view of the X-ray. One chain was tested at a time. For the slack chain and non-slack chain, each end of the chain was secured to the end of a bolt using electrical tape with 12 mm of extra thread between the end beads and the bolts. The single bead chain was also suspended from each end, with the resin bead centered. It was positioned with a total length of thread between the two bolts that is equivalent to that of the slack and non-slack chains (about 15 cm).

### 2.3. Density balancing

The density of the bead chain was tuned to allow for neutral buoyancy within the fluidized bed. Through testing various materials, we found that an effective density of $\sim 0.4\;\text{g}/{\text{cm}}^{3}$ was ideal for allowing an object to remain in motion in the bed without floating to the top or sinking to the bottom. The density of the resin used was 1.23 g/cm^3^. Since poppy seeds cannot fit through the opening in the beads, the inside can be made hollow to decrease the effective density of the beads. The thickness of the walls can be tuned to target the desired density. In these calculations, the mass and volume of the thread is assumed to be negligible.

### 2.4. Discrete element method (DEM) simulation

We used the open-source software package LAMMPS [12] to develop a DEM simulation of the bead chain. To create models of polymers, many simulations rely on Langevin dynamics [13], which includes bonding forces, a viscous damping force, and an excitation force proportional to temperature to model thermal noise. However, because of the size of the system, creating noise on the scale of the excitation of the beads would require non-physical temperatures. For this reason, we used an Verlet integrator [14,15] to model this system with an NVE canonical ensemble, and created randomly generated forces every time step to emulate the noise of the system without requiring a temperature dependence. This approach was intended to be analogous to Langevin dynamics but allow for meaningful forces on a macroscopic scale.

Spherical particles with diameters 1.5 mm and 6 mm were used to model the crimp and the resin beads, respectively (Figure 4A). We used linear and angular restoring force bonds between beads aiming to reproduce the chain behavior in the simulation environment. To model the strings on either end of the chain, we used bonds that produce no force below a threshold and a linear restoring force above it. The anchors, fixed point boundary conditions generated to represent the ends of the string, were placed 101 mm apart. The chain has a total length of 125 mm to match with experiments. The parameters defining the beads, crimps, and bonds used in the simulations are given in Table 1. In addition to these bonds, we include a viscous damping force to emulate Langevin dynamics as described previously. This damping force was tuned such that the mean speed of the beads in simulation was similar to that in experiment (Figure 4C). We apply randomly generated excitation forces on each bead, leading to random variations in the granular temperature of particles, as described in Equation 4. The direction and magnitude distribution of the excitation forces were drawn from either Gaussian and exponential distributions (Figure 4B). The simulation was run for 400,000 timesteps, and data was recorded after 100,000 timesteps to allow the system to reach dynamic equilibrium. We expect Gaussian distributed forces to describe the typical environment of DNA and other polymers in free solution, while the exponentially distributed forces represented are used to represent the velocity distribution of particles in a typical fluidized bed [16]. The bond equations, constraints, and the external forces acting on the chain are given as follows:

(1) $${\vec{F}}_{\text{linear }}=-k_{l}\left(R-\left(r_{b}+r_{t}\right)\right)\overset{ˆ}{r}$$

where ${\vec{F}}_{\text{linear }}$ is the restoring force of the linear bonds, $k_{l}$ is a constant, R is the distance between the centers of two adjacent particles, $r_{b}$ is the radius of the bead, and $r_{t}$ is the radius of the tracer crimp, and $\overset{ˆ}{r}$ is the unit vector pointing to the adjacent particle (Figure 4 A). This acts as a linear restoring force with an equilibrium distance of $\left(r_{b}\right.\left.+r_{t}\right)$.

(2) $${\vec{F}}_{\text{angular }}=k_{a}(\theta -\pi )\overset{ˆ}{\theta }$$

where ${\vec{F}}_{\text{angular }}$ is the restoring force of the angular bonds and θ is the relative angle formed by the centers of three adjacent beads as seen in Figure 4 A, and $\overset{ˆ}{\theta }$ is the angular unit vector. This acts as an linear restoring force on the angle, θ, with an equilibrium length of π.

(3) $${\vec{F}}_{\text{string }}=\left\{\begin{array}{ll} 0 & \ell \leq \ell_{0}\\ -k_{s}\left(\ell -\ell_{0}\right)\overset{ˆ}{\ell } & \ell >\ell_{0} \end{array}\right.$$

where ${\vec{F}}_{\text{string }}$ is the restoring force of linear bonds at the endpoints of the bead chain, $k_{s}$ is a constant, ℓ is the distance from the anchor to the bead at the endpoint, $\ell_{0}$ is the length of the string, and $\overset{ˆ}{l}$ is the unit vector pointing from the anchor to the endpoint bead. This acts as a linear restoring force with an equilibrium distance of $\ell_{0}$ when the distance is greater than the slack of the simulated string, and provides no force when the distance is less.

As an approximation of dissipative forces within the system, we applied a damping force on each bead proportional to the velocity of the particle. This force was calibrated to match the persistence length of bends in the simulation to those of the experiment.

(4) $${\vec{F}}_{\text{resistive }}=-k_{v}\vec{v}$$

where ${\vec{F}}_{\text{resistive }}$ is the force associated with damping, $k_{\nu }$ is a constant, and $\vec{v}$ is the instantaneous velocity of each particle (including both beads and crimps). This acts as a damping force proportional to velocity.

The excitation force corresponding to the thermal noise, ${\vec{F}}_{\text{kick }}$, applied to each bead at each timestep is calculated as follows:

(5) $${\vec{F}}_{\text{kick }}=k_{r}P_{x}\overset{ˆ}{x}+k_{r}P_{y}\overset{ˆ}{y}+k_{r}P_{z}\overset{ˆ}{z}$$

Here, $k_{r}$ is the constant maximum magnitude of the force, $\left(\overset{ˆ}{x},\overset{ˆ}{y},\overset{ˆ}{z}\right)$ are the unit vectors in Cartesian coordinates, and $P_{i}$ is a random number drawn from a distribution function for $i=x,y,z\cdot {\vec{F}}_{\text{kick }}$ is the sum of all the forces calculated for x,y, and z directions, each with its own random number $P_{i}$. In the Gaussian case, $P_{i}$ is drawn from a normal distribution, and in the exponential case it is drawn from the function below:

(6) $$P_{i,\text{ exp }}=\left\{\begin{array}{ll} -\text{log}\left(U_{1}(0,1)\right) & U_{2}(0,1)\leq 0.5\\ \text{log}\left(U_{1}(0,1)\right) & U_{2}(0,1)>0.5 \end{array}\right.$$

where $U_{1,2}(0,1)$ are independent random numbers drawn from a uniform distribution between 0 and $1.U_{2}$, in particular, determines the direction, which can be positive or negative with equal probability. In both cases, random numbers are updated every timestep $\left(1\text{*}10^{-4}\;\text{s}\right)$. Plots of these distributions are shown in Figure 4B.

## 3. Methods

### 3.1. Fluidization process

After the desired chain was secured to the apparatus, the fluidization process began. To ensure a level, loosely packed granular surface, the bed was briefly fluidized by increasing the voltage to the blower while holding the chain out of the bed. Then the chain was laid down to rest on the granular surface.

At the start of each trial, we used a video recording software (OBS) to capture the X-ray output at 30fps. The anemometer reading was also recorded throughout the experiment via an NI USB DAQ-6009 which measured its output signal. The voltage to the blower was increased manually using the Variac until the anemometer reading stabilized at 0.55 m/s, which is above the minimum fluidization velocity for the bed (estimated previously to be $U_{f}\approx 0.32\;\text{m}/\text{s}$), defined as the superficial gas velocity at which the drag force of the upward moving gas through the porous medium becomes equal to the weight of the particles in the bed [17]. At this velocity, sustained movement of the chain was visible. The air velocity for trials was chosen to ensure desired behavior of the bead chain. Motion of the crimps needed to be large enough to represent molecular motion without being so fast as to cause excessive motion blur in the X-ray videos. At velocities just near the minimum fluidization velocity, the bead chain floats on top of the poppy seeds with slight motion. At slightly higher velocities, the chain sinks until the beads rest in a catenary-curve-like shape with little movement. As flow velocity is further increased, the chain becomes immersed in the bed with sustained motion. Once the air speed stabilizes at 0.55 m/s, recording continues for as long as desired or until the air speed is no longer steady. Once the X-ray recording finishes, the blower voltage is turned off and the bead chain settles underneath the granular surface. The chain is then retrieved and the terrain is briefly fluidized to reset the grains to a loosely packed state.

### 3.2. Particle tracking

**FIGURE 2:**
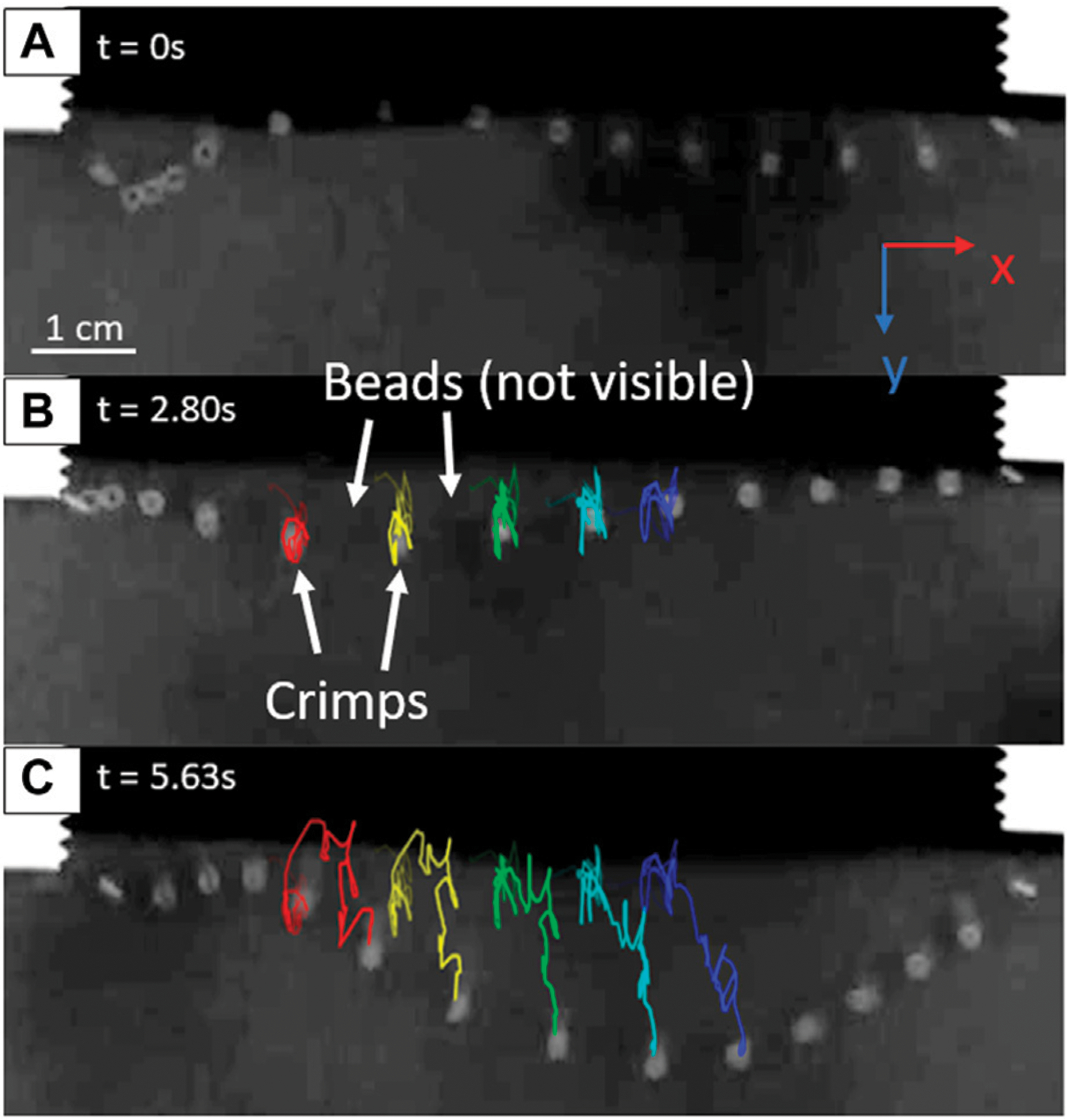
Example images from of a tracked video for the high-slack chain (3.4 mm). (**A**) Initial frame of the recording, where fluidization is already active and agitating the bead chain. (**B**) A frame showing the crimp beads’ tracks up to 2.80 s after the initiation of recording (**C**) Tracks of the crimp beads after 5.63 s

Trackmate, a plugin in the FIJI installation of ImageJ, is used to track the position of the metal tracer crimp beads in the videos [18]. Before tracking, the video is cropped, and the contrast is enhanced using FIJI. If necessary, the brightness or levels may also be adjusted to improve detections. The video is inverted such that the beads are lighter on a darker background. The Linear Assignment Problem (LAP) tracker is used, with parameters such as threshold, gap closing distance, and expected spot diameter adjusted for each video to yield the most accurate detections. The trajectories of the middle five tracer crimp beads (far from the constrained ends) are analyzed (Figure 2). The resin beads are not able to be tracked due to their lower radioopacity causing a lack of contrast with the background when immersed in poppy seeds. In frames where motion blur prevented Trackmate from finding certain beads, manual detections are performed. For some videos, track editing was necessary to ensure that each track corresponded to a specific tracer bead. The particle positions are then exported from Trackmate and used to calculate the x and y velocities of the middle five crimp beads. The z velocity (movement into and out of the plane of the detector) was omitted due to the capture being two dimensional.

## 4. Results

**FIGURE 3:**
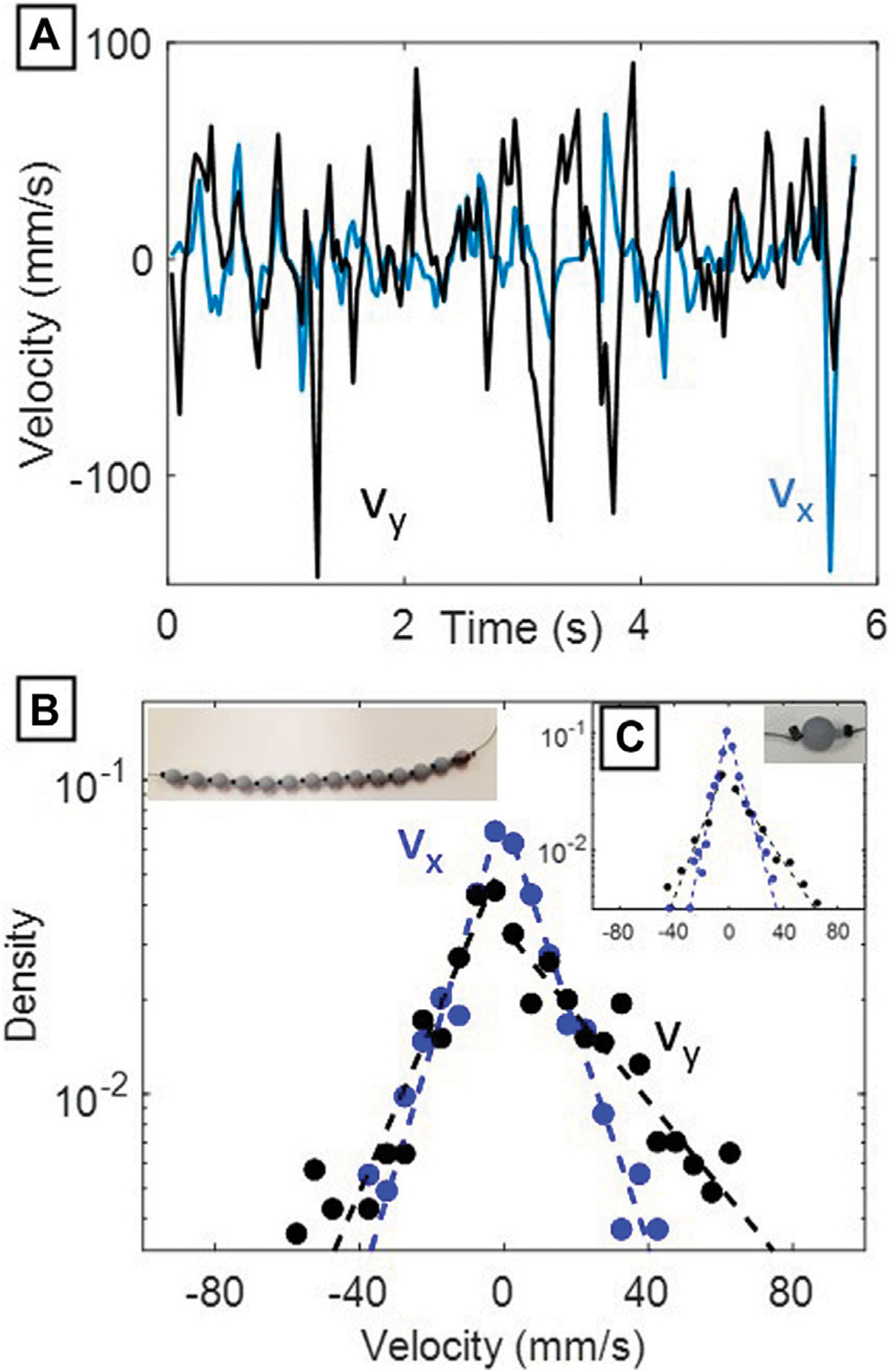
Results from physical experiments. (**A**) x and y velocities over time of the middle bead in the low-slack (3.4 mm) chain. (**B**) Semi-log velocity probability densities for the center 5 beads from the full-chain experiments. Dashed lines indicate best exponential fits calculated separately for positive and negative velocities. The image in the top left shows the original configuration of the beads used in this experiment.(**C**) Semi-log velocity probability densities from the single bead experiments (image shown in top right). Dashed lines indicate best exponential fits calculated separately for positive and negative velocities.

The distributions of the x and y velocities of the tracer beads in the physical experiment appeared exponential on a semi-log plot (Figure 3). The distributions had greater asymmetry in $v_{y}$ (parallel to gravity and the airflow direction), while the transverse distribution of velocities $v_{x}$ was symmetrical due to spatial symmetry in the experimental configuration. In addition, the distributions were generally wider in $v_{y}$ than in $v_{x}$ axis, likely because the beads are less able to move longitudinally (along the string) than perpendicularly to the string. This effect was reproduced in simulation. The single bead did not follow a different type of distribution than the longer bead chains, implying that the presence of adjacent beads on the same string did not influence the exponential nature of the distribution.

**FIGURE 4:**
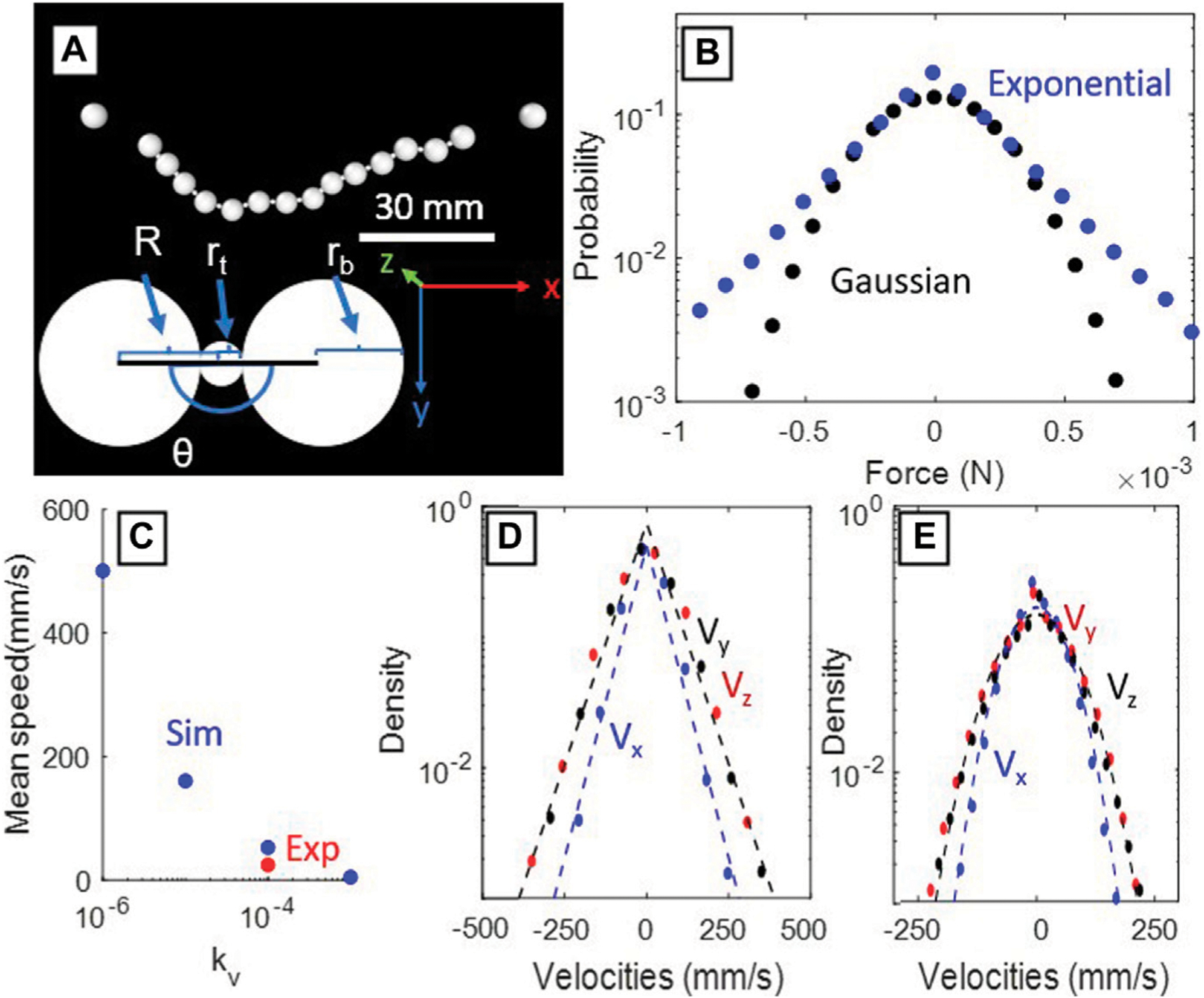
Results from the DEM simulations. (**A**) Example frame from the simulation intended to replicate the conditions of the experimental setup. (**B**) The random forcing distributions in each dimension for Gaussian (black) and exponential (blue) distributions. (**C**) A calibration plot of the drag coefficient of the system. The blue points are the mean speeds of the beads in simulation, while the red is the mean speeds of our experimental trial. This plot allows calibration the viscous damping force in simulation. (**D**) The resulting velocity distributions when the particles are subjected to exponential kicks. Dashed lines indicate best exponential fits.(**E**) The resulting velocity distributions when the particles are subjected to Gaussian kicks. Dashed lines indicate best Gaussian fits.

Previous studies of individual particles in homogeneous fluidized beds have observed stretched exponential velocity distributions $\left(P(v)\sim \text{exp}\left(-\alpha v^{\beta }\right)\right.$) with β≈1 (as in Figure 4B) in three dimensional systems [16]. Deviations from a pure Gaussian distribution (β=2) for each velocity component will also shift the system away from the Maxwell-Boltzmann statistics of an ideal kinetic gas [16,19]. The additional constraints of being in a chain may further deviate the particles’ velocities from a Gaussian distribution, thus we wanted to separate the external forcing and kinematic constraint conditions. To independently test constraint induced changes of the chain’s observed kinematics (Figure 3), we turned to the DEM simulations to generate arbitrary forcing distributions on the bead.

The DEM simulations demonstrate that if the distributions of the forces acting on the beads are exponential, the resulting velocity distribution of the beads themselves will be exponential (β=1) as seen in Figure 4D, while Gaussian distributed forces will generate Gaussian distributed velocity distributions (β=2) as seen in Figure 4E. This result in combination with our single bead experimental results (Figure 3C) shows that the resultant velocity distributions from the bead chain approximate the shape of the forcing distribution. The experimental velocity distributions thus are longer-tailed than Gaussian velocity distributions that would result from Brownian motion. The kinematic constraint of being on a chain did not significantly effect the velocity distributions, thus the dominant factor modulating β will be the forcing mechanism used for bed agitation.

## 5. Discussion and conclusion

Macroscale bead chains in controllable fluidized beds offer a promising physical 3D analog for solute microscopic polymer chains like DNA. Various fluidization and agitation mechanisms of the bed can adjust the velocity distributions of the grains and bead chains. Possible experimental improvements would include multi-plane X-ray capturing techniques and improved fluidized bed airflow control. Another challenge in this study was the density balancing for the buoyant bead chain such that it would not sink or be expelled from the bed over longer time scales. 3D printing provides a promising method for creating macro-scale models of molecules, as it allows for both rapid manufacture and precise tuning of the chained beads’ effective density.

For tracking tracer particles (e.g., the bead crimps in our study), FIJI Trackmate can serve as a suitable method, but contrast enhancing techniques like CLAHE and watershed segmentation [20] could serve to better track and distinguish the rapid motion of constrained fluidized tracer particles in future studies. Many trials had infrequent high-velocity bead movements, representing the tails of our measured velocity distributions. These high-velocity events often occurred in only a single frame and were difficult to resolve, possibly leading to inaccuracies in the experimental distributions. In longer, faster-sampled experiments, we might expect these singleframe, high-velocity outliers to have a much lower probability density than presented in Figure 3. We were limited by the type of X-ray detector used, having only a small field of view and a low frame rate of 30fps. Future work could focus on increasing the length of chains and capturing a faster frame rate (as in [21]) to resolve motion blur issues. In addition, the magnitude of environmental forces in the simulation could be further calibrated by measuring the drift velocity of a single free bead (as in [22]) to correlate the strengths of the fluctuations to the magnitude of the dissipative force.

We hypothesize that in our experiment, the net movement of the bead chain was almost purely driven by the buffeting of fluidized grains from the input airflow and constrained by the strings holding the bead chains. The presence or absence of neighboring beads had an insignificant effect on the motion of either one or many bead(s) on a string, since a single bead had a similar velocity distribution to a full bead chain. The pressure drop in the fluidized bed during airflow is expected to be homogeneous across the bed in the plane normal to the gravity vector, according to the theory of fluidized beds, but this was not measured. However, the actual velocity distributions of the poppy seeds were not uniform in time or space due to bubbling, and are expected to follow an overall exponential distribution [16]. The spread of these velocity distributions are dependent on the geometries of each particular fluidized bed. This possible inhomogeneity in the driving particle velocities (the poppy seeds), coupled with increased constraints near the terminal ends of the bead chain, created a situation where central beads were both the freest to move and affected by the highest speed particles. This supports our result of the resulting exponential velocity distribution we observed (Figure 3B) in the bead chain, that differs from a Gaussian distribution that would arise from Gaussian distributed forces (Figures 4B, E).

For modeling microscopic polymer systems, although this study only modulated the amount of slack on the chain, many other properties could be changed to mimic the size, shape, and stiffness of specific polymers. With resin printing, the size and shape of the beads can easily be modified to explore the effects of different physical properties on the model. For example, by attaching magnets to the bead chains’ ends, we can also pin the chain into loops if the ends meet under fluctuation to mimic protein-mediated DNA looping [23]. We also plan to increase the complexity and size of our bead chain to better model large biopolymers in a more realistic setting. By inserting many interaction sites in the bead chain, we will be able to study folding dynamics of the chain and the emergence of higher-order structures with potential relevance to chromatin. In addition, since the movement of the fluidized grains in this system is induced externally, future experiments may allow the bead chain to map onto studies of constrained passive tracers (analogous to the beads) in active ensembles of thermal particles (analogous to the fluidized grains) [24]. Future studies can also explore a wide range of chain composition and confinement geometry, which will provide insights into the physics of large and flexible biopolymers [25,26] and DNA telomeres in living cells [27].

## Figures and Tables

**TABLE 1:** Parameters used for the DEM simulations.

Property	Value
Bead radius (*r*_*b*_)	3 × 10^−3^ m
Tracer radius (*r*_*t*_)	7.5 × 10^−4^ m
Particle density	5 × 10^−3^ kg/m^3^
String Length (ℓ_0_)	1.2 × 10^−2^ m
Timestep	1 × 10^−4^ s
Length of Simulation	4 × 10^5^ timesteps
Linear Bond Strength (*k*_*l*_)	1 N/m
Angular Bond Strength (*k*_*a*_)	1 × 10^−2^ N/m
String Bond Strength (*k*_*s*_)	5 N/m
Excitation Coefficient (*k*_*r*_)	1 × 10^−2^ N
Damping Coefficient (*k*_*v*_)	1 × 10^−4^ N/(m*s)

## Data Availability

The original contributions presented in the study are included in the article/Supplementary Material, further inquiries can be directed to the corresponding authors.
